# Grazing-incidence small-angle X-ray scattering: application to the study of quantum dot lattices

**DOI:** 10.1107/S0108767311040104

**Published:** 2011-11-11

**Authors:** Maja Buljan, Nikola Radić, Sigrid Bernstorff, Goran Dražić, Iva Bogdanović-Radović, Václav Holý

**Affiliations:** a Ruđer Bošković Institute, Bijenička cesta 54, 10000 Zagreb, Croatia; b Sincrotrone Trieste, SS-14 km 163.5, 34144 Basovizza, Italy; c Jožef Stefan Institute, Jamova cesta 39, 1000 Ljubljana, Slovenia; d Faculty of Mathematics and Physics, Charles University, Ke Karlovu 5, 121 16 Prague, Czech Republic

**Keywords:** grazing-incidence small-angle X-ray scattering, GISAXS, quantum dot lattices, self-assembly

## Abstract

The ordering of quantum dots in three-dimensional quantum dot lattices is investigated by grazing-incidence small-angle X-ray scattering (GISAXS). Theoretical models describing GISAXS intensity distributions for three general classes of lattices of quantum dots are proposed. The classes differ in the type of disorder of the positions of the quantum dots. The models enable full structure determination, including lattice type, lattice parameters, the type and degree of disorder in the quantum dot positions and the distributions of the quantum dot sizes. Applications of the developed models are demonstrated using experimentally measured data from several types of quantum dot lattices formed by a self-assembly process.

## Abstract 2

The modelling of grazing-incidence small-angle X-ray scattering (GISAXS) from three-dimensional quantum dot lattices is described.

## 1. Introduction

Materials containing quantum dots (QDs) have been widely investigated in the last decade because of their interesting size-tunable properties (Alivisatos, 1996 ▶; Bostedt *et al.*, 2004 ▶; Hanson, 2009 ▶) and many potential applications in semiconductor technology and opto-electronic devices (Jabbour & Doderer, 2010 ▶; Ladd *et al.*, 2010 ▶; Konstantatos & Sargent, 2010 ▶). Especially interesting is the production of materials that contain regularly ordered QDs, often called QD lattices. The regular ordering of QDs implies narrowing of the QD size distribution and better control over the QD separations (Buljan, Desnica *et al.*, 2009 ▶ *a*). The applicability of such materials is often based on the quantum confinement effect of carriers (Bostedt *et al.*, 2004 ▶) or on collective effects (Grützmacher *et al.*, 2007 ▶), which are both very sensitive to the arrangement and size properties of the QD system.

Ordered QD systems can be fabricated by various methods. The most usual one is the growth of crystalline multilayers where the lattice mismatch between different layers causes ordering of QDs mediated by the local elastic strain fields (Stangl *et al.*, 2004 ▶) or by colloidal synthesis (Alivisatos, 2000 ▶). Recently it was shown that the production of self-ordered QDs is also feasible in *amorphous* multilayers (Buljan, Desnica *et al.*, 2009 ▶ *a*,*b* ▶; Buljan, Pinto *et al.*, 2010 ▶; Buljan, Grenzer, Keller *et al.*, 2010 ▶). The ordering in such systems was achieved by growth at an elevated substrate temperature, at which an interplay of diffusion and surface morphology mechanisms causes the self-organized growth and formation of three-dimensional QD lattices. Some other recent investigations (Buljan, Bogdanović-Radović *et al.*, 2009 ▶, 2010 ▶, 2011 ▶) demonstrated the formation of long-range-ordered quantum dot arrays in an amorphous matrix by ion beam irradiation. In this growth method the ordering is induced by irradiation of an entirely amorphous multilayer by light ions under oblique incidence.

For the successful development and subsequent application of the methods for the production of well ordered QD arrays, the experimental methods for precise structural characterization of such materials are very important. Real-space imaging techniques like transmission electron micrography (TEM) or atomic force microscopy (AFM) are often used; however, they probe the structure of a limited area of the sample cross section or surface only, so that the statistical relevance of the data might be poor. The advantage of scattering methods in the far-field limit like grazing-incidence small-angle X-ray scattering (GISAXS) is that they yield experimental data with excellent statistics (typically 10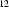 QD in the irradiated volume). This is especially suitable for the analysis of ordered QD systems such as QD lattices, where spatial correlations in QD positions can be easily observed and qualitatively described. On the other hand, scattering methods are indirect, since they measure a reciprocal-space distribution of the scattered intensity and the retrieval of real-space information is not a trivial task. Usually, one has to use a suitable structure model, from which the reciprocal-space distribution of the scattered intensity is simulated and compared to experimental data. Direct methods for the retrieval of the real-space image from data in reciprocal space work only on a single quantum dot, so that they cannot give information relevant for a large dot ensemble (see Pfeifer *et al.*, 2006 ▶ and citations therein).

Up to now GISAXS has been successfully applied to the analysis of many QD systems, and a comprehensive review showing the basic theory of GISAXS and its different applications is given in Renaud *et al.* (2009 ▶). There are several software packages available for the simulation of GISAXS data [*IsGISAXS* (Lazzari, 2002 ▶) and *FitGISAXS* (Babonneau, 2010 ▶)].

In most works published to date, only two-dimensional disordered arrays of nano-sized objects (nanocrystals, quantum dots *etc*.) have been considered (see Renaud *et al.*, 2009 ▶ and references therein) and only very little attention has been paid to a detailed analysis of regularly ordered three-dimensional ensembles of nano-objects. In our previous works (Buljan, Desnica *et al.*, 2009 ▶ *b*; Buljan, Bogdanović-Radović *et al.*, 2010 ▶) we have developed two models for GISAXS characterization of three-dimensional lattices of nanocrystals. However a detailed and comprehensive formulation of a variety of possible three-dimensional ordered arrays of quantum dots is still missing. In this paper we formulate several theoretical models of the positions of quantum dots in three-dimensional quantum dot lattices. Two approaches are used, namely the short-range-order (SRO) model and the long-range-order (LRO) model. Starting from one-dimensional SRO and LRO models we formulate a two-dimensional SRO model of the dot positions similar to the well known ideal paracrystal model (IPM, see Eads & Millane, 2001 ▶). Then, based on this two-dimensional model, we develop three distinct three-dimensional SRO/LRO models of the dot positions. We use the models of the dot positions for the simulation of the reciprocal-space distribution of the intensity scattered in a GISAXS experiment. Each structure model is accompanied by an experimental example. The application of the models allows determination of the type of QD lattice, lattice parameters, the parameters of the position disorder, as well as the average size of the QDs and their size distribution. The models are especially suitable for the description of QD lattices grown by a self-organization process in multilayers or homogeneous thick films; however they can be applied to any QD lattice that fulfils the model constraints.

The paper is organized as follows. In §2 we show several experimental examples of QD lattices and discuss their structural properties. We also demonstrate that different structural models have to be used for their description. The main part of the paper is contained in §3, where the one-dimensional, two-dimensional and three-dimensional structural models of the QD lattices are developed. This section also contains examples where the theoretical simulations of the GISAXS intensity distributions are compared with experimental data. The limitations of the developed models and some important notes for their successful application in the structural analysis are given in §4. The conclusions are given in §5.

## 2. Three-dimensional QD lattices – structural overview

**Figure 1:**
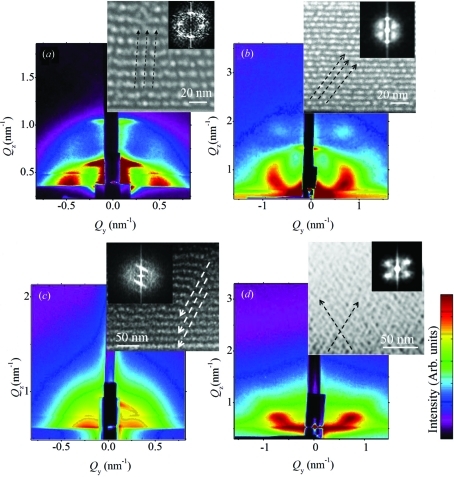
GISAXS intensity distributions and corresponding STEM images shown in insets measured on different films containing Ge QD lattices in amorphous matrices. (*a*) (Ge+SiO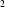)/SiO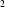 multilayer deposited on a flat substrate at 773 K, and annealed at 1073 K after the deposition. (*b*) (Ge+Al
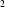
O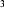)/Al
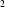
O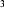 multilayer deposited on a flat substrate at 773 K. (*c*) (Ge+SiO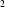)/SiO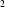 multilayer deposited at room temperature and irradiated with 3 MeV O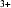 ions. The multilayer was annealed at 1073 K after the irradiation. (*d*) Ge+Al
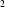
O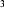 continuous thick film deposited on a flat substrate at 773 K.

In this section we describe several types of QD lattices, which serve as representative experimental examples of systems with various types of ordering of the dot positions. The first system is formed by self-organized growth of a (Ge+SiO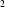)/SiO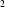 multilayer on a flat substrate (Buljan, Desnica *et al.*, 2009 ▶ *a*,*b* ▶). The QDs are formed within the layers of the multilayer, so the vertical components of the QD lattice vectors obey long-range ordering, induced by the multilayer periodicity. The regular three-dimensional ordering of the Ge quantum dots formed is achieved during deposition at 773 K and it is induced by an interplay of the surface morphology effect and diffusion-mediated nucleation. The resulting lattice of QDs has rhombohedral structure and consists of small domains randomly azimuthally rotated around the normal to the multilayer surface. A scanning tunnelling electron microscopy (STEM) image of the system and the corresponding GISAXS map are shown in Fig. 1 ▶(*a*). In the STEM image, a weak regularity in the dot positions can be observed; however the number of QDs in the depicted area is too small for a reliable determination of the degree of ordering. In contrast, because of a very large number of coherently irradiated dots, the ordering is very clearly visible in the GISAXS map – a regular ordering gives rise to strong satellite intensity maxima. A similar system, but with a better degree of ordering, is represented by a (Ge+Al
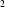
O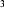)/Al
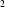
O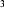 multilayer (Buljan, Radić *et al.*, 2011 ▶). The GISAXS map of such a QD lattice and the corresponding STEM image are shown in Fig. 1 ▶(*b*).

A further example considers a QD system formed by ion beam irradiation of a fully amorphous (Ge+SiO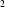)/SiO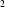 multilayer (Buljan, Bogdanović-Radović *et al.*, 2009 ▶, 2010 ▶, 2011 ▶). The angle of the irradiation was 60° with respect to the multilayer surface. The irradiation causes ordering of QDs in chains along the irradiation direction. The lateral positions of the chains obey a two-dimensional SRO model; however the lateral positions of the dots in a given chain are ordered according to a one-dimensional LRO model. The vertical dot positions are almost perfectly periodic, since they follow exactly the multilayer periodicity. GISAXS and STEM images of this system are shown in Fig. 1 ▶(*c*). Ordering of the QDs along the chains in the irradiation direction is visible in the STEM image, while the presence of the dot chains is the reason for strong tilted intensity maxima (‘Bragg sheets’) in the GISAXS map. The in-plane correlation of the positions of the chains causes additional lateral satellites.

The last example demonstrating an ordering obtained in a single continuous Ge+Al
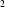
O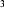 layer is shown in Fig. 1 ▶(*d*). Even if no multilayer was deposited, a regularly ordered QD system was formed during the film growth. Therefore the main difference from the previous examples is that the vertical positions of the QDs in a QD lattice are not pre-determined like in the multilayer case. Thus, the vertical positions of the QDs obey the SRO model as well. Details of the driving force for the QD ordering in this system can be found in Buljan, Pinto *et al.* (2010 ▶). More examples of QD lattices and corresponding GISAXS maps can be found in Buljan, Grenzer, Keller *et al.* (2010 ▶), Buljan, Grenzer, Holý *et al.* (2010 ▶) and Pinto *et al.* (2011 ▶).

The GISAXS intensity distributions were measured at the small-angle X-ray scattering beamline of the synchrotron Elettra, Trieste, Italy, using a photon energy of 8 keV, and a two-dimensional image-plate detector. The detector was perpendicular to the probing sample and almost perpendicular to the incoming X-ray beam. The scattered radiation was collected for a constant incidence angle slightly above the critical angle of total external reflection of the investigated films. STEM images were taken with a JEOL2010F microscope, operated at 200 kV and equipped with a field-emission gun and a high-angle annular dark-field detector (HAADF) for *Z*-contrast imaging.

All the examples listed above present ordered QD arrays, which differ not only in the degree of ordering, but also in the ordering model. The structure of the multilayer stack, irradiation effects and/or self-assembly features have different effects on the type of QD ordering. In particular, the QDs may follow an LRO-type ordering model along some direction and SRO along the other ones. In addition, the degree of disorder may be different in different directions. These simple examples demonstrate the rich variety of various orderings and the importance of a proper formulation of the ordering model. In the next section, we present a theory describing three models of dot ordering corresponding to the experimental examples presented above, and we show the respective simulated GISAXS intensity distributions.

## 3. Quantum dot ordering models and simulation of the scattered intensity

The distribution of the intensity in reciprocal space scattered in a GISAXS experiment can be calculated by the distorted-wave Born approximation (DWBA). In this approach one divides the sample into two parts – a non-disturbed system and the disturbance. The scattering from the non-disturbed system is calculated exactly (*i.e.* using the multiple-scattering dynamical theory), whereas the disturbance scatters only kinematically. This approach is very frequently used; however its validity has to be discussed and confirmed in any particular system. Generally speaking, the DWBA approach is applicable if multiple scattering from the disturbance can be neglected. In the case of quantum dots arranged in a three-dimensional matrix embedded in a semi-infinite medium, one usually considers the medium as the non-disturbed system and the ensemble of the quantum dots as the disturbance.

In the following we assume that the dots are fully buried in an amorphous semi-infinite substrate with an ideally flat surface (*i.e.* the influence of the surface roughness is neglected). The reciprocal-space distribution of the wave scattered from the substrate exhibits an infinitely narrow rod-like maximum along the surface normal (crystal truncation rod, CTR) and the intensity distribution along the CTR is determined by the specular reflectivity of the substrate.

In the following, we neglect this wave and consider only the wave scattered from the dots. The reciprocal-space distribution of the wave scattered from the dots is 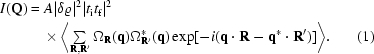
In this formula 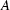 is a constant, 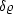 is the difference in the electron densities of the dot material and the surrounding matrix, 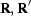 are position vectors of the dots, 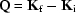 is the scattering vector (the difference of the wavevectors of the scattered and incident beams), 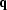 is the complex scattering vector corrected to refraction at the vacuum–substrate interface (for details see Renaud *et al.*, 2009 ▶) and
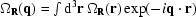
is the Fourier transformation of the shape function 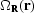 of a dot occurring in position 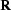; the shape function is unity in the dot volume and zero outside it. 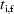 are the Fresnel transmittivities of the substrate surface corresponding to the primary and scattered waves, respectively; the factor 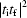 exhibits a maximum (so-called Yoneda wing) if the incidence angle 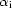 and/or the exit angle 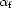 equal the critical angle 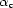 of total external reflection.

The 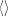 brackets in equation (1) denote the averaging over the positions and shapes of the quantum dots. In order to calculate this averaging, one has to assume how the dot sizes are connected with their positions. In the literature, two limiting approaches can be found (Renaud *et al.*, 2009 ▶). The decoupling approximation (DA) assumes that the sizes of the dots are not statistically correlated with their positions (Guinier, 1963 ▶). Strictly speaking, this approximation is valid only in very diluted systems; usually it is reasonable to assume that the distance between larger dots is on average larger than between smaller dots. The local monodisperse approximation (LMA) assumes that the sample is divided into domains, each domain containing dots of a given size and given distribution of the distances (Pedersen, 1994 ▶; Renaud *et al.*, 2009 ▶). In each domain one calculates the average over the dot positions and finally the averaging over the domains is carried out. In this paper we will restrict ourselves to the DA only.

Within the DA, the averaging indicated in equation (1) is straightforward. After some algebra one obtains 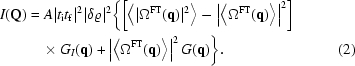
Here we have denoted 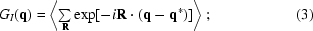
this function equals the number 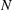 of the QDs if we neglect the imaginary part of the scattering vector 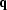. The function 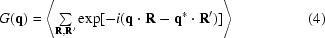
is the correlation function of the dot positions; the averaging here is performed only over the dot positions. In the following we denote 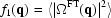 and 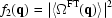.

The main goal of this paper is to formulate physically relevant models of the positions of the quantum dots, from which we can calculate the correlation function 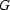. As we emphasized in §1, both SRO and LRO approaches are used. Within SRO, the position of a given dot is affected only by the positions of the neighbouring dots, while LRO assumes that the dots randomly deviate from pre-defined periodic ideal dot positions. In the following, we will derive the correlation functions for one-dimensional and two-dimensional dot arrays arranged within the SRO and LRO models, and finally we present the correlation function of a three-dimensional dot ensemble; for this case we will use a combination of the SRO and LRO models.

### 3.1. One-dimensional SRO model

Let us start with a one-dimensional chain of quantum dots along the 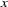 axis and we index the dots by the integer index 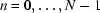. The position of the dot with index 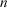 with respect to the origin is denoted by 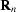 which can be expressed as a sum of random connection vectors 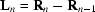, 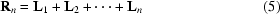
or in terms of basis vectors 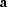 of one-dimensional ideal (undisturbed) lattice and deviation vectors 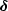 
               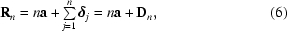
where 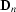 denotes the total deviation of a dot with index 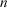 from its ideal position. The mean value of the connection vectors 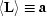, *i.e.* 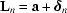. We assume that 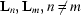 are statistically independent.

**Figure 2:**
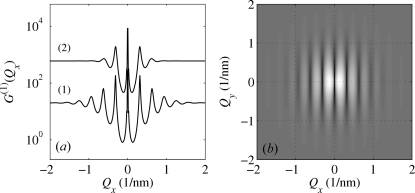
(*a*) Correlation function of the one-dimensional SRO model plotted along the 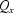 axis parallel to the dot chain (see text for the chain parameters). The simulations were performed for a fixed mean number 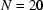 of the dots and the same mean separation 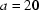
 nm; 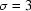
 nm for line (1) and 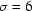
 nm for line (2). (*b*) Correlation function 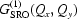 as a function of two components 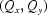 calculated with the same parameters as for line (1); the dot chain is oriented along the 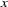 axis.

A direct calculation of equation (4) yields the one-dimensional correlation function in the form 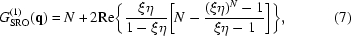
where 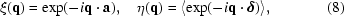
and we have neglected absorption effects, so 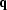 = 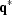. Absorption will be introduced in the three-dimensional model. In equation (7) 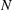 denotes the number of the coherently irradiated dots; if this number is very large (*i.e.* if the mean dot distance is much smaller than the size of the coherently irradiated sample surface), one can use the limiting expression for 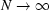: 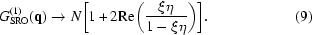
The function 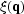 contains the undisturbed positions of the dots, while the function 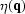 depends on the statistical distribution of the deviation vectors 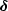. We have assumed that the components 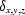 of the random deviation 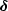 are normally distributed with zero mean and root mean square (r.m.s.) dispersion 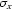,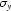 and 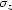, 
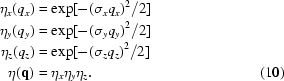
Fig. 2 ▶ presents examples of the calculated correlation functions for 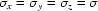. In panel (*a*) of this figure we plotted the values of 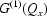 along the 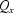 axis parallel to 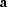. The correlation function exhibits maxima (satellites) in the points 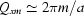, where 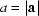 and 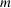 is an integer (satellite order). The full width at half-maximum (FWHM) of the zero satellite is 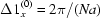; in the limiting case in equation (7) the central peak is infinitely narrow (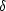-like). For finite 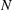, the central maximum is accompanied by tiny fringes with the period of 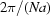. Since the degree of coherence of the primary beam usually continuously decreases from unity to zero, these fringes are not observed and in the following they are removed by averaging the correlation function over various *N*’s. This averaging does not affect the shape of the non-zero satellites. The FWHMs of the non-zero satellites depend almost quadratically on the satellite order 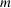.

Fig. 2 ▶(*b*) displays the one-dimensional correlation function 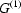 as a function of two components 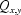 of the scattering vector. In the reciprocal 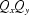 plane the correlation function exhibits a streak along the 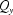 axis, with increasing 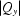 the streaks become broader and weaker. Here we have neglected refraction and absorption to keep the focus on the ordering properties. Thus, for this case, 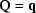. Refraction and absorption effects will be introduced later in three-dimensional models.

### 3.2. One-dimensional LRO model

A one-dimensional system of QDs can be described by an LRO model if the positions of QDs fluctuate independently around their *pre-defined* (ideal) positions. Thus, within the LRO model, the position 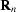 of the 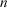
th dot can be expressed as 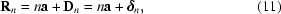
where random vectors 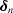 describe the deviation of the dot from its ideal position. Within the SRO model, the position of the dot with index 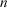 was defined with respect to the position of the dot with index 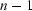, so the total deviation from the undisturbed position increases with 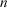. Thus, the main difference between SRO and LRO models is the total deviation vector of the dot 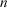 with respect to the origin: 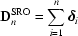 for the SRO model while 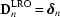 for the LRO model.

Assuming that vectors 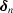 are statistically independent we obtain the correlation function for the LRO model, 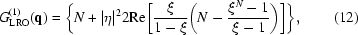
where 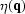 and 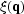 are defined in equation (8).

**Figure 3:**
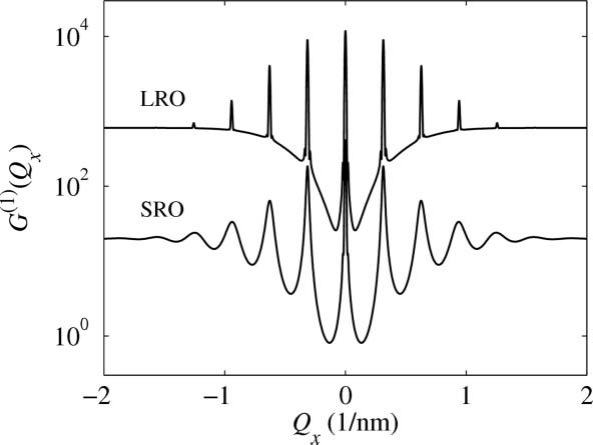
Comparison of correlation functions of one-dimensional SRO and LRO models calculated with the same parameters as in Fig. 2 ▶.

Fig. 3 ▶ compares the correlation function of one-dimensional chains of QDs arranged in LRO and SRO models. Analogously to the SRO model we assumed that the random deviations 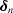 have zero average values and their components are normally distributed, while different components of 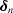 are statistically independent. In contrast to the SRO model, the widths of the correlation peaks in the LRO do not depend on the r.m.s. deviation 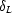 and they are inversely proportional to the size 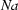 of the coherently irradiated chain. By increasing the disorder in the dot positions, the diffuse part of the correlation function between the maxima increases.

### 3.3. Two-dimensional models

The construction of a physically sound two-dimensional SRO model is not a straightforward task. One possible approach (the IPM; Eads & Millane, 2001 ▶) assumes that each dot is labelled by two indexes 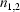 and its position vector can be written as 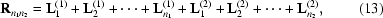

**Figure 4:**
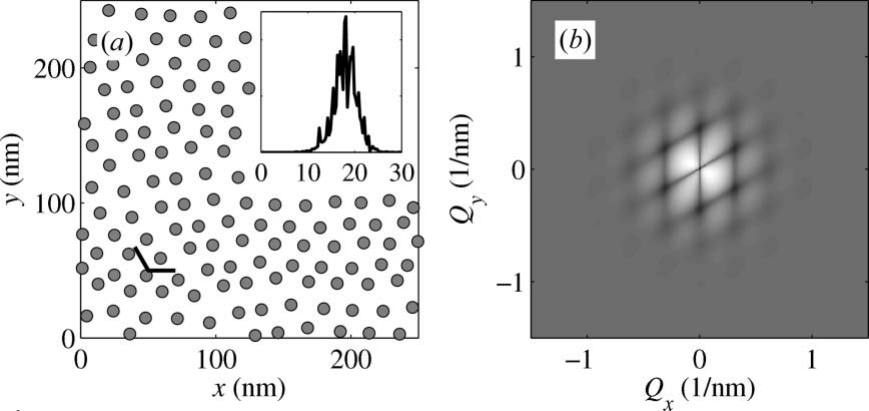
(*a*) Positions of the dots randomly generated using the two-dimensional ideal paracrystal model (IPM). The inset displays the histogram of the nearest dot distances, the pair of short black lines denote the vectors 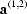. The parameters of the correlation are described in the text. (*b*) The two-dimensional correlation function of the IPM calculated with the same parameters as in panel (*a*).

               *i.e.* two types of the connection vectors 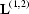 are assumed with the mean values 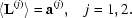
Therefore, the IPM assumes that the dots occupy the points of a disordered two-dimensional lattice with the lattice vectors 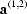, 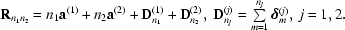
After simple calculation we obtain the following expression for the two-dimensional correlation function, 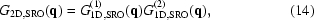
where 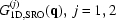 are the one-dimensional correlation functions described in equation (7), in which the functions 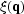 and 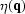 are replaced by 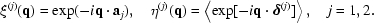
Fig. 4 ▶(*a*) shows the positions of the dots generated randomly using the IPM and normal distribution of the deviations 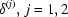; in the simulation we used the values 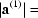 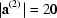
 nm and 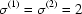
 nm. The corresponding correlation function is plotted in Fig. 4 ▶(*b*). The satellite maxima of the correlation function lie in the points of a lattice reciprocal to the lattice generated by the vectors 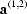; the FWHMs of the maxima increase with the satellite orders.

**Figure 5:**
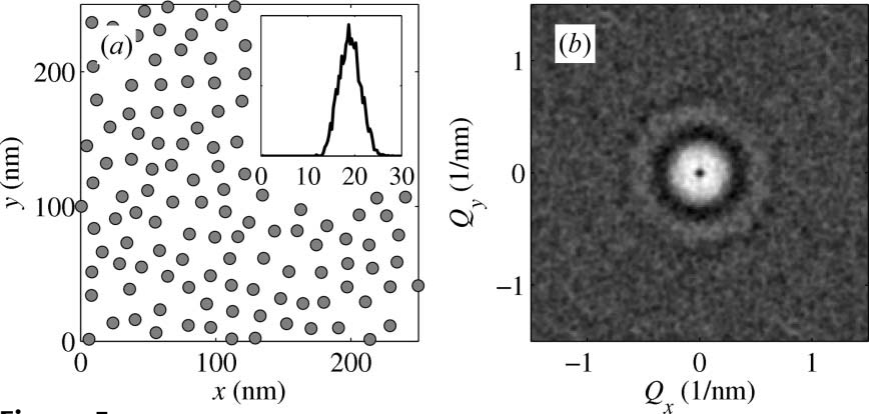
(*a*) Positions of quantum dots randomly generated using a given distribution of nearest distance and an accept–reject method; we used the same mean distance and the r.m.s. deviation as in Fig. 4 ▶. The inset shows the actual distribution of the nearest distances determined from the generated dot positions. (*b*) The two-dimensional correlation function obtained from the dot positions shown in panel (*a*).

The IPM is not fully applicable if the dots are created by a self-organization process resulting in a random lattice, since the IPM assumes the existence of an *a priori* defined ideal lattice with the basis vectors 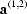. This is illustrated in Fig. 5 ▶, where we have plotted the positions of the dots generated randomly assuming that the random nearest dot distances obey the Gamma distribution with the given mean 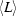 and given r.m.s. dispersion 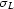. The simulation has been carried out using the Monte Carlo (MC) accept–reject sampling method described by Robert & Casella (2004 ▶).

Comparing Figs. 4 ▶(*a*) and 5 ▶(*a*) it is obvious that, in contrast to the IPM, the array of randomly generated dots does not exhibit any pre-defined lattice directions, in spite of the fact that the distributions of the nearest dot distances are very similar (see the insets in Figs. 4 ▶ *a* and 5 ▶ *a*). The correlation function of the randomly generated array of dots is isotropic (see Fig. 5 ▶ *b*) and no distinct satellite maxima in reciprocal-lattice points are visible.

**Figure 6:**
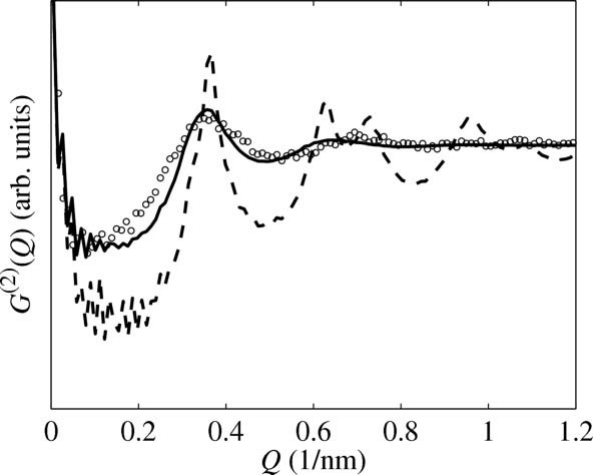
The radial correlation function of the two-dimensional SRO model obtained by numerical Monte Carlo method (dots) using 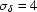
 nm, and the azimuthally averaged correlation functions of the IPM model with 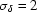
 nm (dashed line) and 4 nm (unbroken line).

In Fig. 6 ▶ we compare the radial profile 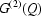 of this correlation function with the radial profile of the correlation function 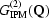 (plotted in Fig. 4 ▶ *b*) averaged over all azimuthal directions of the vector 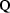. The dashed line denotes the azimuthally averaged function 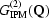 which was calculated for the same value 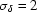
 nm as that used by the MC simulations in Fig. 5 ▶(*a*); obviously the maxima in this correlation function are much narrower than those following from the MC simulation. In order to get a good match of both radial correlation functions, we have to increase the 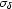 value of the IPM model to 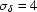
 nm (unbroken line). From Fig. 6 ▶ it follows that the correlation function of the IPM azimuthally averaged over all directions of the scattering vector 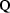 is a good approximation of the correlation function of a two-dimensional SRO model generated by an MC simulation, in which the directions of the connection vectors 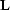 are isotropically distributed; however, one has to use an approximately two times larger r.m.s. dispersion of the dot distances in the IPM model.

### 3.4. Three-dimensional models

In the previous sections we constructed the one- and two-dimensional SRO models as well as an LRO model of the positions of quantum dots and we calculated the corresponding correlation function. The next step, *i.e.* the definition of a three-dimensional model, depends much on the mechanism of the ordering of the quantum dots during their nucleation and growth. In the following, we formulate three various three-dimensional models realized by different experimental recipes and compare the theoretical descriptions with experimental results.

For all systems we assume that the quantum dots create a disordered three-dimensional lattice with the averaged basis vector 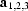. Each dot is labelled by three indexes 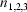 and its position is given by 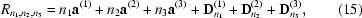
where 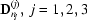 are the random displacement vectors, describing the deviation of the dot position from the ideal position from the origin corresponding to the basis vectors 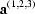.

**Figure 7:**
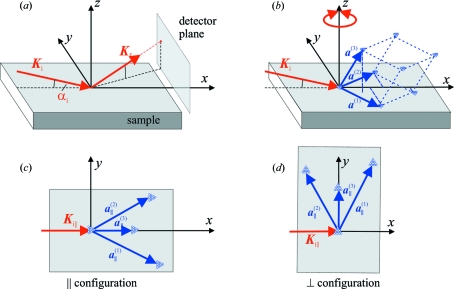
The geometry of the GISAXS experiment. (*a*) The orientation of the primary and scattered X-ray beams with the wavevectors 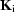 and 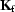, respectively. The plane of incidence is 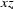, the detector plane is parallel to the 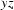 plane. (*b*) The orientation of the basis vectors 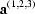 of the dot lattice. The vectors 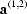 lie in the 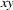 plane parallel to the sample surface. The circular arrow indicates possible averaging over the azimuthal orientations of the vector set 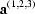, keeping constant the angles between the vectors. (*c*) and (*d*) show the parallel 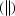 and perpendicular 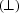 configurations, in which the vector 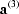 lies in the 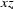 and 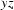 planes, respectively.

The SRO and LRO models differ in the definition of the displacement vectors as was shown in §§3.1 and 3.2: 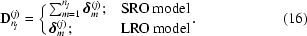
The geometry used for the description and modelling of GISAXS intensity distributions is schematically shown in Fig. 7 ▶. The primary X-ray beam lies in the 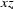 plane (plane of incidence) and makes a small angle 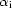 (angle of incidence) with the 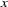 axis (Fig. 7 ▶ *a*). All experimental GISAXS maps were taken with 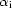 = 0.2°, *i.e.* very close to the critical angle 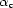 of total external reflection. In the actual experimental arrangement the detector plane was perpendicular to the primary beam; however, for the sake of simplicity we calculate the intensity distribution in the reciprocal 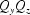 plane perpendicular to the sample surface. The distortion of the intensity map due to the angle 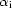 of the detector plane with the 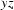 plane is negligible.

The vectors 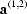 lie in the plane parallel to the substrate (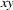 plane), while the direction of the vector 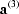 corresponds to the direction of the correlation of the positions of the dots belonging to different periods of the multilayer. The 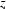 component of 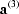 [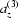] corresponds to the multilayer period. Thus, the coordinates of the basis vectors 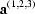 are 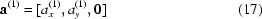

               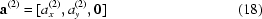

               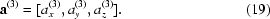
The choice of the basis vectors is based on the growth process of the samples. The diffusion and growth properties are usually similar in the plane parallel to the substrate, while they are different in the growth direction (assumed perpendicular to the substrate). However, the models developed are generally valid for any choice of the basis vectors. We will use two configurations in the simulations of GISAXS intensity distributions, namely assuming that (i) the probing beam is parallel (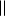, Fig. 7 ▶ *b*) and (ii) perpendicular (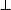, Fig. 7 ▶ *c*) to the common plane of 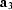 and the surface normal.

The absorption effects are included in the three-dimensional model *via* the imaginary part of the complex scattering vector 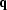. For the chosen geometry, 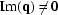 only for the 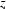 component of 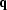, while the parallel components are real and equal to those in vacuum 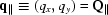. To keep the formulas as simple as possible, we neglect the absorption in the distances comparable to the deviations 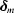 of the dots from their ideal positions. Then, the functions 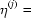 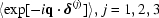 defined in the previous section contain only the real part 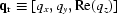 of the scattering vector 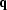.

The total intensity [equation (1)] in the three-dimensional case is given by
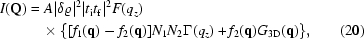
where 
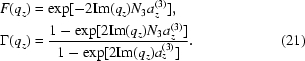

               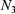 is the number of the dots along the basis vector 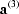 and 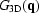 is given by the product of three one-dimensional correlation functions. The functions 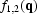 are defined in §3.

In the three-dimensional models discussed later we will treat separately the 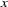, 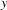 and 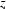 components of the random vectors 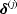 to have the generally valid formulas. This is necessary because deviations around ideal positions are not necessarily isotropic; their r.m.s. deviations may be different in different directions, for example in the case of nucleation on pre-patterned substrates. Another reason is that the ordering type may be different for different components of the same basis vector (SRO or LRO), as in the case of the multilayer stack which is described by the LRO model, while the basis vector 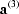 is not perpendicular to the multilayer surface. All these cases will be shown in the specific models given below. Thus we deal in total with three components of three deviation vectors (nine in total), and we assume that the components 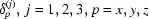 are statistically independent with zero means and r.m.s. dispersions 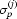. Therefore the functions 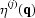, (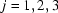) can be written as a product of three components: 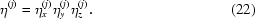
The components are given by 
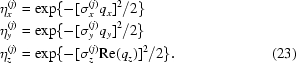
In the following we consider three specific cases (models) differing in the type of QD ordering.

### 3.5. Model 1

Model 1 describes a system of QDs with the same type of ordering along all three average basis vectors [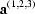]. If the QD positions along all basis vectors obey SRO ordering, this model is suitable for the description of QD systems formed by a self-assembly process with no external constraints. Such systems may be realized by arrays of QDs formed by self-ordered growth in thick homogeneous layers (Buljan, Pinto *et al.*, 2010 ▶) or in multilayers where the layer sequence can be described by the SRO model.

The correlation function 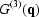 for this case is a generalization of the two-dimensional SRO ideal paracrystal model, *i.e.* it is a product of three one-dimensional SRO correlation functions: 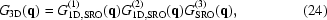
where the 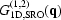 are given by equation (7) and 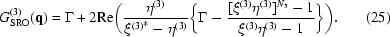
Here, 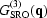 differs slightly from 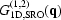 because absorption effects are included in it *via* the imaginary part of the 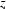 component of the scattering vector 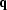.

**Figure 8:**
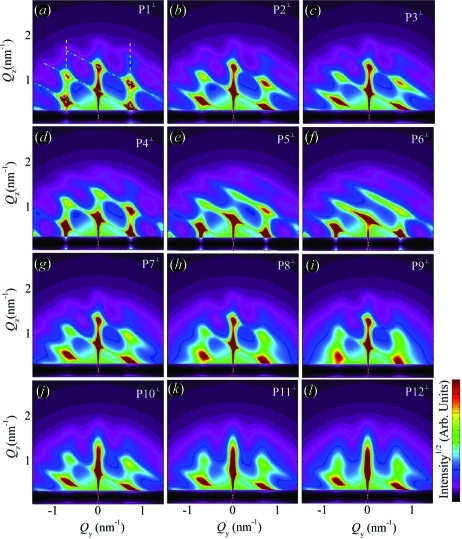
Simulations of two-dimensional intensity maps obtained with model 1 for various values of the disorder parameters. The results for the 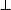 geometry [the probing beam perpendicular to the in-plane component of the basis vector 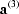] are shown. We assumed 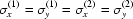 and 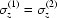. (*a*)–(*c*) Influence of 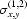. The dashed lines parallel to the 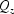 axis indicate the sheets caused by the in-plane correlation of the QD positions. The correlation of the dot position in different layers gives rise to tilted sheets indicated by dash–dotted lines. (*d*)–(*f*) Influence of 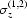. (*g*)–(*i*) Influence of 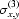. (*j*)–(*l*) Influence of 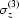. The symbols P1–P12 denote the sets of disorder parameters given in Table 1 ▶. The QDs are assumed to be spherical.

Using correlation function 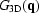 and equation (20), we have simulated the two-dimensional GISAXS intensity distributions. The simulations are shown in Fig. 8 ▶. The simulations are performed for various parameters of the disorder. The QDs are assumed to be spherical and arranged in a rhombohedral lattice with the basis vectors given in Table 1 ▶, along with the parameters of the disorder and dot sizes. Two types of intensity sheets (indicated by the lines in Fig. 8 ▶ *a*) may be distinguished in the GISAXS simulations shown in Fig. 8 ▶. The first type are the sheets (streaks) placed parallel to the 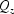 axis. These sheets are the consequence of the correlation of the QD positions within the plane parallel to the substrate (in-plane correlation). They become broader and weaker with increasing 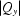, and their FWHMs also increase with growing in-plane components of the in-plane disorder, *i.e.* with increasing 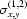. This is visible in Figs.  ▶8(*a*), 8 ▶(*b*), 8 ▶(*c*). The effect of the increase in the vertical component of the in-plane disorder [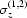] causes a decrease in intensity and a lateral broadening of the sheets with an increase in 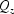 (see Figs. 8 ▶ *d*, 8 ▶ *e*, 8 ▶ *f*).

The second type of sheets are the tilted ones. They appear as a result of the correlation in the QD positions corresponding to different layers. The influence of the increase in the lateral [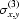] and vertical [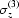] disorder on this type of sheet is illustrated in Figs. 8 ▶(*g*), 8 ▶(*h*), 8 ▶(*i*) and 8 ▶(*j*), 8 ▶(*k*), 8 ▶(*l*), respectively. The increase in the lateral disorder causes a broadening and weakening of the correlation peaks in the 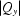 direction, while the increase in the vertical disorder broadens the sheets along 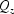. In summary, for model 1, in which all the disorder components are described by SRO, all correlation peaks broaden with the increase in the degree of disorder.

**Figure 9:**
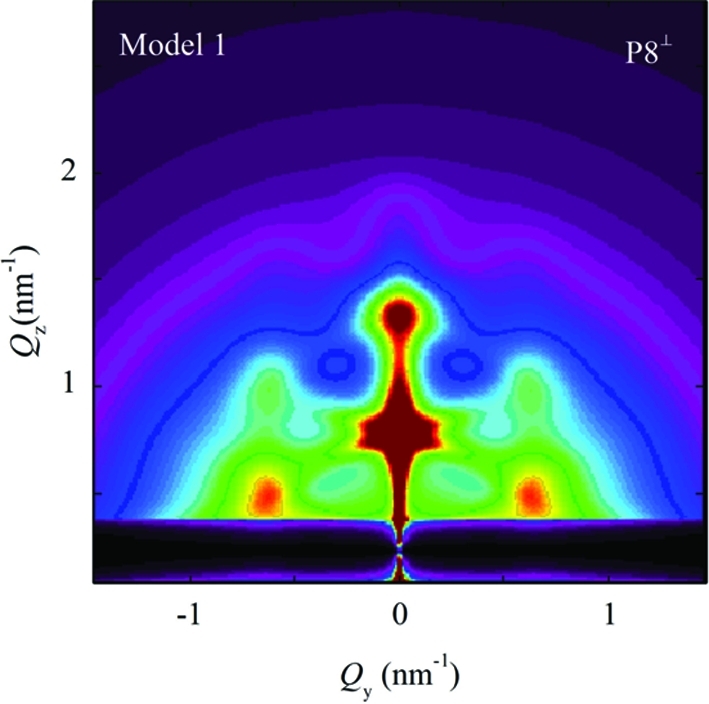
Simulation of two-dimensional GISAXS intensity map obtained using model 1 and azimuthal averaging for the set P8 of the disorder parameters. The intensity scale is the same as in Fig. 8 ▶.

The simulations shown in Fig. 8 ▶ are obtained for the perpendicular geometry with no averaging of the azimuthal directions of 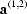. As stated previously (see §3.3), this case may be successfully used for systems where some pre-defined direction of the basis vectors exists. But, for systems with no pre-defined direction or with domains randomly rotated around the normal to the surface, the azimuthal averaging (over all rotations of basis vectors around the 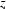 axis) should be performed (see Fig. 7 ▶). An example showing simulation of the azimuthally averaged intensity distribution (using the parameter set P8) is shown in Fig. 9 ▶. The influences of the parameters on the peak profiles follow the same rules as in the non-averaged system (Fig. 8 ▶).

An example of the application of this model to self-assembly of Ge quantum dots in continuous thick Al
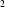
O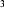 film is shown in the next section.

### 3.6. Example 1: self-assembly of Ge quantum dots in an alumina matrix

Here we present an example showing the application of model 1 for the description of Ge QD lattices produced by magnetron sputtering deposition of a continuous Ge+Al
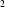
O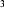 layer at 773 K on a flat substrate. Owing to the elevated deposition temperature QDs form during the layer growth. The dots formed during the deposition affect the shape of the growing surface, which incites a self-organization process during the layer growth. The result of the deposition is the formation of domains of QDs that are ordered in a three-dimensional tetragonal lattice.

**Figure 10:**
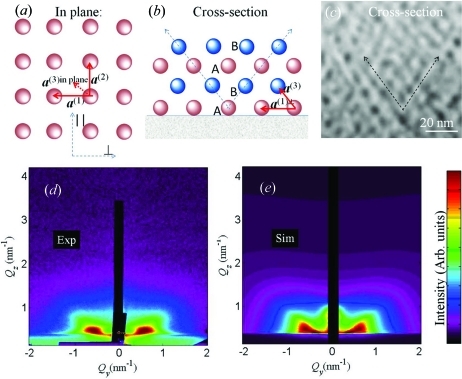
Schematic views of the Ge QD lattice formed in a continuous Ge+Al
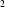
O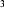 layer by a self-assembly process. The lattice is described by basis vectors 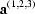; (*a*) and (*b*) depict the plane parallel and perpendicular to the surface, respectively. (*c*) STEM image of the film cross section. The surface is parallel to the bottom edge of the image. (*d*) Experimentally measured and (*e*) simulated GISAXS maps. The parameters of the simulations are given in Table 2 ▶.

The formed dot lattice is schematically presented in Figs. 10 ▶(*a*), 10 ▶(*b*) while the experimentally measured STEM cross section of the film is shown in Fig. 10 ▶(*c*). The domains are randomly rotated with respect to the surface normal. More details about the origins of self-assembly in this kind of film are given in Buljan, Pinto *et al.* (2010 ▶). The nature of the deposition process indicates that the ordering in all directions can be described by the SRO model: the substrate used for the deposition is isotropic and flat and it actually does not influence significantly the QD ordering. On the other hand, a single continuous film is deposited, so there is also no reason for a long-range ordering in a direction perpendicular to the surface, which would be the case for a regular multilayer.

Experimentally measured and simulated GISAXS maps of this sample are shown in Figs. 10 ▶(*d*) and 10 ▶(*e*), respectively. The positions of the lateral maxima in the measured map do not depend on the azimuthal direction of the primary X-ray beam. This means that the regular ordering appears in domains that are randomly azimuthally rotated. The same follows from the STEM images of the film. Therefore, the simulation of the experimentally measured map was performed by averaging of equation (20) over all azimuthal orientations of the basis vectors. The parameters used for the simulation are given in Table 2 ▶. In the fitting procedure of the GISAXS data we assumed that some components of the r.m.s. deviations 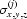 are equal because of the sample symmetry, *i.e.* 
               
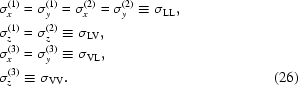
The indexes L and V in equation (26) are used to describe disorder of the longitudinal (parallel to the substrate) and vertical (perpendicular to the substrate) components of the basis vectors, respectively. The first index refers to the basis vector described, and the second one to the deviation vector. Thus, σ_LV_ describes the vertical deviation of the in-plane basis vectors **a** ^(1)^ and **a** ^(2)^. Model 1 is valid for this sample, since the parameters obtained are in good agreement with those from STEM, which are also given in Table 2 ▶.

### 3.7. Model 2

Model 2 describes a three-dimensional QD array where the QDs are ordered according to the long-range-order model along the basis vector 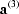, and the short-range ordering occurs in the other directions. This model is suitable for the description of QDs arranged in a multilayer, where the long-range ordering along 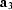 is induced by a process defining ‘ideal’, *i.e.* non-disturbed, positions of the dots. Such a process may be ion beam irradiation of a multilayer (Buljan, Bogdanović-Radović *et al.*, 2010 ▶, 2011 ▶), or regular patterning of the substrate in one direction. In Buljan, Bogdanović-Radović *et al.* (2010 ▶) we have shown the ordering of the positions of Ge quantum dots in a (Ge+SiO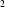)/SiO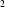 multilayer achieved by a post-growth irradiation of a multilayer by ion beam. The points where the tracks of individual ions cross the Ge-rich layers represent the ideal positions of the Ge quantum dots. Therefore, the position of the 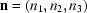-th dot can be expressed by equation (15), where 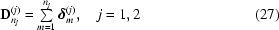
are the random lateral displacements of the dots obeying the SRO model, and the random displacements 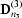 are defined with respect to the ‘ideal’ positions 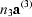. In the multilayer sample mentioned above, the vertical component 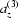 equals the multilayer period and the direction of the basis vector 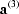 is defined by the direction of the irradiating ions.

**Figure 11:**
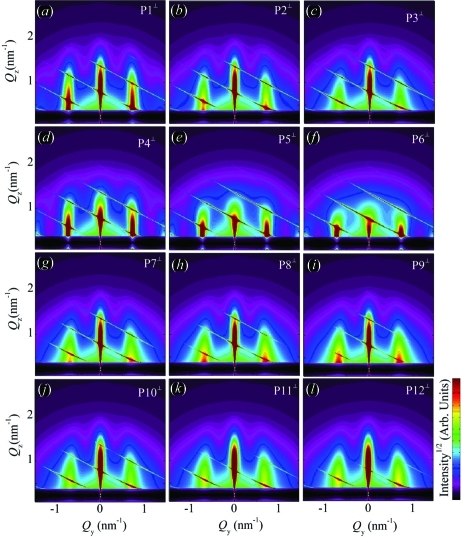
Simulations of GISAXS intensity distribution maps obtained from QD lattices described by model 2. The simulations show the dependence of the intensity distribution on the degree of disorder. (*a*)–(*c*) Influence of 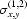. (*d*)–(*f*) Influence of 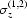. (*g*)–(*i*) Influence of 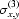. (*j*)–(*l*) Influence of 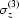. The sets of the disorder and QD lattice parameters are denoted by P1–P12 and given in Table 1 ▶. The QDs are assumed to be spherical.

In this case, the correlation function equals a product of two one-dimensional SRO correlation functions and one one-dimensional LRO correlation function, 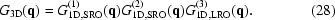
Functions 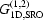 are given by equation (7), 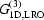 is the one-dimensional correlation function of the LRO model including absorption [see also equation (12)], 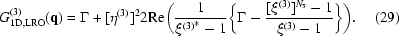
Fig. 11 ▶ shows simulated GISAXS maps obtained for the same sets of the disorder parameters P1–P12 as in model 1.

The properties of the lateral correlation sheets (stemming from the in-plane correlations) are the same as those for model 1: the sheets broaden in the 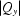 direction with the increase in 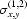 and along 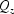 with 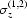. However, the properties of the correlation sheets coming from the ordering along 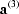 are different from those shown for model 1. The most important feature is the width of these sheets, which is constant in the direction perpendicular to the direction of 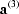. The increase in the disorder parameters 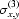 and 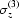 causes a decrease in their intensities in the directions of 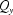 and 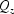, respectively, but the widths remain constant. This feature is a consequence of the LRO model assumed along 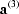.

**Figure 12:**
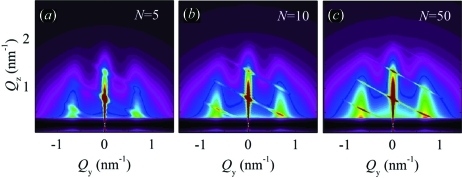
Simulations of two-dimensional GISAXS intensity maps obtained using model 2 with various values of 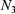 indicated in the figure, and the set of disorder parameters P8 given in Table 1 ▶. The intensity scale is the same as in Fig. 11 ▶.

However, the width of the sheets increases with decreasing 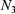. This effect is illustrated in Fig. 12 ▶.

**Figure 13:**
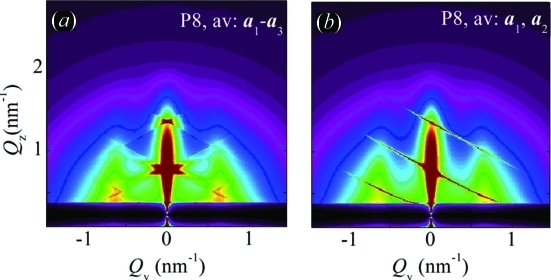
Simulations of two-dimensional GISAXS intensity maps obtained with model 2 and azimuthal averaging for the set of disorder parameters P8. (*a*) The azimuthal directions of all vectors 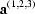 are included in the azimuthal averaging; (*b*) only the azimuthal directions of the vectors 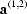 are averaged, the direction of 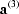 is fixed. Perpendicular geometry is shown in (*b*). The intensity scale is the same as in Fig. 11 ▶.

Azimuthal averaging for all three basis vectors in the systems described by model 2 (see Fig. 13 ▶ *a*) is not common, since the LRO model assumes the existence of a pre-defined direction (given by the basis vector 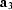, in our case). However, within this model, the azimuthal averaging can be carried out with respect to the basis vectors 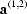 only. Therefore, the QDs make LRO-ordered chains along 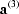, but the ordering of the chains in the plane parallel to the substrate should be averaged over all azimuthal orientations of 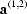. This case is shown in Fig. 13 ▶(*b*). The lateral sheets parallel to the 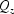 axis, visible in Fig. 13 ▶, are the consequence of the in-plane correlations of the QD positions. The width of these sheets is slightly broader when compared with the non-averaged case (see Fig. 11 ▶ *h*). This is expected because we ‘see’ different projections of basis vectors 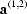 due to the azimuthal averaging.

The application of model 2 to the analysis of GISAXS maps experimentally measured on the ordered QD array produced by ion beam irradiation is given in the next section.

### 3.8. Example 2: quantum dot lattices formed by ion beam irradiation

**Figure 14:**
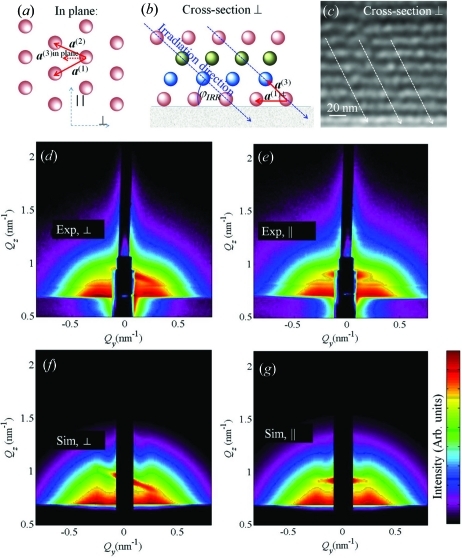
(*a*), (*b*) Schematic views of the structure of the QD lattice formed by ion beam irradiation of a (Ge+SiO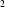)/SiO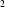 multilayer followed by annealing. The QD lattice is described by the basis vectors 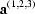, blue dashed arrows indicate the irradiation direction. (*c*) STEM cross section of the film. (*d*), (*e*) GISAXS maps measured on the same film parallel and perpendicular to the irradiation plane, respectively. (*f*), (*g*) GISAXS simulations obtained using model 2, corresponding to the measured maps in panels (*d*), (*e*).

An example of a QD arrangement that can be described by model 2 is a (Ge+SiO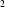)/SiO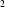 multilayer irradiated by oxygen ions and subsequently annealed. Owing to the ion beam irradiation, QDs are formed along the traces of individual ions (Buljan, Bogdanović-Radović *et al.*, 2009 ▶, 2010 ▶, 2011 ▶). We choose the basis vector 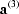 to be directed along the traces. The positions of the traces in the lateral 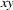 plane can be described by the SRO model and for the description of the lateral positions of the traces we use the basis vectors 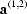. The total intensity is obtained after azimuthal averaging of the basis vectors 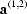, while the third basis vector 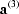 is kept fixed. The schematical view of the QD arrangement is shown in Figs. 14 ▶(*a*), 14 ▶(*b*), while the STEM image of the film cross section is shown in Fig. 14 ▶(*c*). The GISAXS maps of the same system measured in parallel 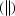 and perpendicular 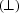 geometries are shown in Figs. 14 ▶(*d*) and 14 ▶(*e*), respectively.

In the perpendicular configuration, the sheets stemming from the ordering along 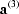 are perpendicular to 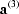, *i.e.* they are tilted; the tilt angle with respect to the surface normal equals the angle of 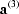 with the surface. In the parallel configuration, the sheets are parallel to 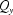.

The simulations of the measured GISAXS maps are shown in Figs. 14 ▶(*f*), 14 ▶(*g*). The simulations are performed using the azimuthal averaging of the basis vectors 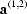 (see Fig. 13 ▶ *b*). We have fitted the model parameters to the experimentally measured GISAXS maps. The resulting parameters are in very good agreement with those obtained from the STEM image (see the numerical values in Table 2 ▶).

### 3.9. Model 3

Model 3 is designed for the description of QD arrays where QDs are long-range ordered along a direction different from the direction of any basis vector (say in the 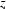 direction), while the ordering in all other directions obeys the SRO model. Thus, the arrangement along the basis vectors is of a ‘mixed’ nature – the lateral components of the random displacements 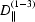 obey the SRO model, while the vertical components 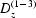 are arranged according to the LRO model. Model 3 is applicable if the dots occur in a multilayer, where the vertical periodicity of the multilayer imposes the ‘ideal’ vertical components of the dot position vectors. The position vector of a dot with indexes 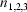 is therefore 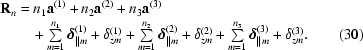
The correlation function for this model is 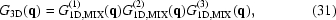
where 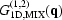 are given by 
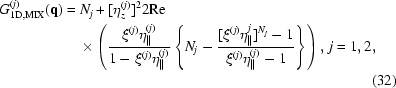
and
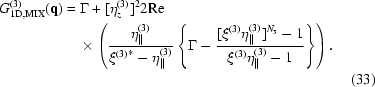
Here we have denoted 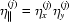.

**Figure 15:**
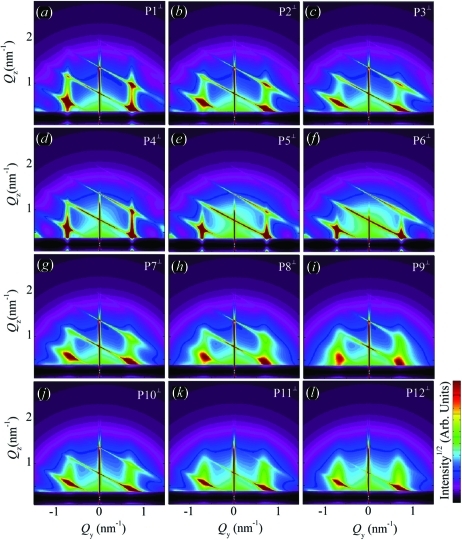
Simulations of the GISAXS intensity maps with model 3; the results in the 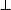 geometry are shown. The simulations show the dependence of the intensity distribution on the degree of disorder. (*a*)–(*c*) Influence of 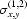. (*d*)–(*f*) Influence of 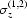. (*g*)–(*i*) Influence of 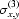. (*j*)–(*l*) Influence of 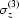. As in the previous GISAXS maps, the symbols P1–P12 denote the parameter sets in Table 1 ▶.

The simulations of the GISAXS maps for various disorder degrees are shown in Fig. 15 ▶. The behaviour of the sheets caused by the in-plane ordering is the same as in models 1 and 2. However, the width of the sheets corresponding to the correlation of the positions in different layers is different. In accordance with the ‘mixed’ nature of the correlation function [equation (33)], the width of the streaks increases along 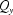. However the width in the 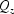 direction is constant, but the intensity decreases if the disorder parameter 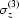 increases. However, if 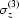 is sufficiently small, models 1 and 3 yield very similar results.

**Figure 16:**
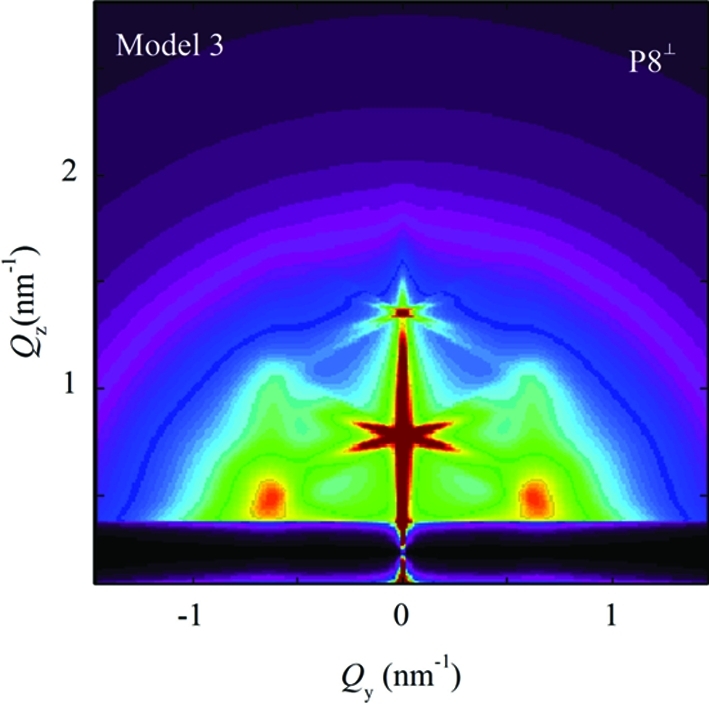
Simulated GISAXS intensity map from the QD lattices described by model 3 after azimuthal averaging. The set P8 of the disorder parameters is used. The intensity scale is the same as in Fig. 15 ▶.

The influence of the azimuthal averaging on the results of model 3 is shown in Fig. 16 ▶. Similarly to model 1, azimuthal averaging makes the GISAXS intensity distribution sym­metric with respect to the 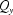 = 0 axis. Also, it is not sensitive to the azimuthal orientation of the probing beam with respect to the sample. This is expected due to averaging over all possible azimuthal orientations. The peaks visible in Fig. 16 ▶ are broader than for the non-averaged case (Fig. 15 ▶).

An example showing the application of this model to a QD lattice produced by self-ordered growth on a flat substrate is given in the next section.

### 3.10. Example 3: quantum dot lattices formed by self-assembly on a flat substrate

**Figure 17:**
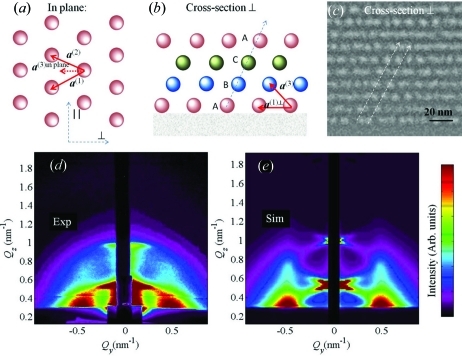
(*a*), (*b*) Schematic views of the QD arrangement in the QD lattice formed during self-assembled growth of a (Ge+SiO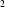)/SiO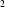 multilayer. The lattice formed has a three-dimensional rhombohedral structure with the 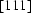 axis perpendicular to the sample surface. It is described by basis vectors 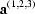. (*c*) STEM image showing ordering within a small domain. (*d*), (*e*) Measured and simulated GISAXS maps, respectively. The parameters of simulation are given in Table 2 ▶.

Here we show the application of model 3 for the simulation of the GISAXS maps of QD lattices formed by self-assembled growth of Ge QDs in an amorphous SiO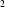 matrix. The samples are produced by magnetron sputtering of 20 (Ge+SiO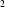)/SiO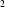 bi­layers on a flat Si(111) substrate (Buljan, Desnica *et al.*, 2009 ▶ *b*). The multilayer is periodic, *i.e.* the vertical distances of the QDs follow the long-range-ordering model. The deposition was performed at an elevated substrate temperature making possible the self-assembly of the dots. The resulting lattices of quantum dots have a rhombohedral face-centred-cubic-like structure. The ordered regions appear in domains randomly rotated around the surface normal. The arrangement of the QDs in a domain is schematically shown in Figs. 17 ▶(*a*), 17 ▶(*b*), while the STEM measurement of the film cross section is shown in Fig. 17 ▶(*c*). The measured GISAXS intensity distribution is shown in Fig. 17 ▶(*d*). The intensity distributions are not sensitive to the azimuthal direction of the X-ray probing beam (see Fig. 7 ▶).

The analysis of the measured maps is performed using model 3. Additionally we have performed an azimuthal averaging of the calculated intensity, to include the effect of randomly oriented domains. The parameters of the QD lattices and sizes of the QDs, obtained by fitting of the measured GISAXS maps to the theoretical maps, are shown in Table 2 ▶. Examples of measured and simulated GISAXS maps, using the parameters obtained by the fit, are shown in Figs. 17 ▶(*d*) and 17 ▶(*e*), respectively.

Model 3 has also been successfully applied to the description of the ordering of Ge quantum dots deposited on rippled Si substrates (Buljan, Grenzer, Keller *et al.*, 2010 ▶), SiGe multilayers (Pinto *et al.*, 2011 ▶) and ordering of Ge QDs in an Al
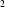
O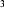 matrix (Buljan, Radić *et al.*, 2011 ▶).

## 4. Discussion, limitations, surface and interface effects

In the previous sections we have developed models for the description of GISAXS intensity distributions for the various types of QD lattices. We have applied these models for the analysis of a variety of experimentally realized systems and we have shown that the obtained structural parameters are in very good agreement with the STEM results. Here we compare different models and consider several points that should be taken into account in the analysis of measured GISAXS maps.

**Figure 18:**
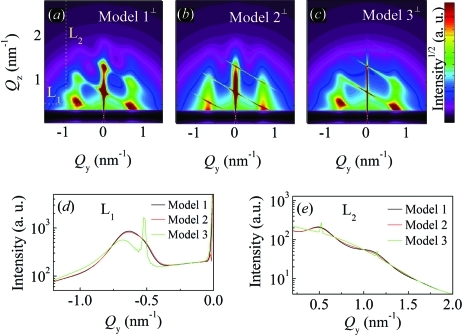
(*a*)–(*c*) Comparison of simulated GISAXS maps obtained with different models using the set of parameters P8. (*d*), (*e*) Comparison of one-dimensional intensity profiles taken along lines 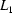 and 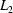, respectively [indicated in (*a*)] for models 1–3. Perpendicular geometry is shown.

A comparison of GISAXS intensity distributions for various models developed above is shown in Fig. 18 ▶. From the figure it is evident that the same set of parameters yields different GISAXS intensity distributions for different models. Therefore, for a proper description of the system and analysis of GISAXS data, it is very important to choose the correct model type. If the model is incorrectly chosen, the parameters obtained by the fit can lead to non-realistic parameters or the fitting process cannot simulate well the experimentally measured spectra. This problem can be avoided if the properties of the growth procedure of the QD lattice are known. Then, LRO or SRO can be expected along a particular spatial direction. For example, if a periodic multilayer is deposited and the deposition process is precisely controlled, so the same conditions are valid for each layer of a multilayer, the LRO is expected in the growth direction. However, if a similar multilayer is deposited, but the deposition conditions are not precisely controlled for each layer, then the expected ordering will be SRO. Of course, the processes like self-assembly usually yield SRO of the QDs, while regular (LRO) surface patterning yields LRO of the QDs (Holý *et al.*, 2009 ▶).

If the type of QD ordering cannot be estimated based on the deposition procedure, then the properties of the correlation peaks stemming from LRO or SRO should be considered. The most important difference between LRO and SRO is the width of the correlation peaks, which increases with the peak order for SRO, while it is constant for LRO (for the one-dimensional case). The type of ordering is then determined based on the properties of correlation peaks.

There are also several other points that should be taken into account, which relate to possible limitations of the model. The first one has already been mentioned earlier in this paper, and it concerns the description of two-dimensional or three-dimensional SRO systems. The commonly applied ideal paracrystal model imposes the existence of preferred orientations that usually do not exist in real systems. We have shown that this problem can be overcome by averaging over different azimuthal orientations of the lattice.

The second problem is the effect of the overall shape of the QD lattice for which the simulation is performed. The QD lattice is described by basis vectors 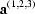 and in the simulation we assumed given numbers 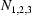 of the unit cells along the basis vectors. Therefore, the dot lattice domain has the shape of a parallelepiped with given directions of the edges. This rather non-physical shape of the lattice domain affects the GISAXS intensity distribution, but this effect is significant only in the very close vicinity of the origin of reciprocal space. Thus, the simulated GISAXS intensity is not correct only for very small values of 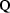. This is easily visible in the experimental examples shown above – the most significant differences between the experimental data and simulations appear only in the vicinity of the specular plane and for very small values of 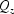.

**Figure 19:**
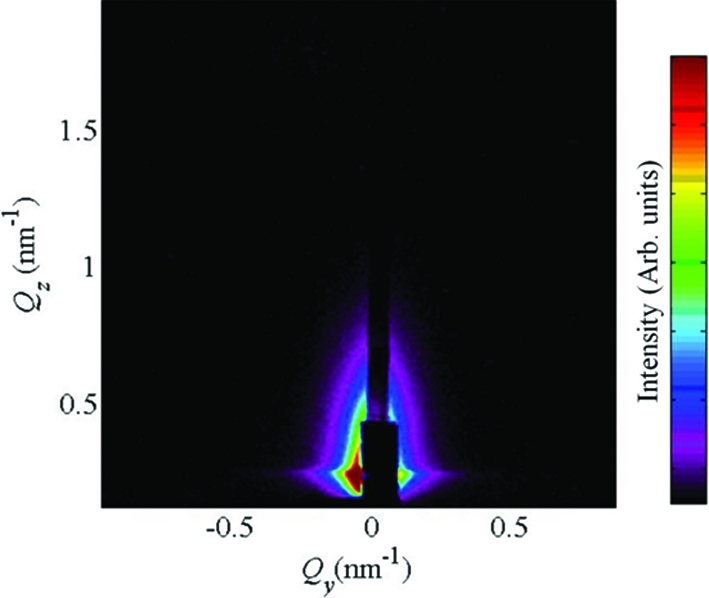
The contribution of surface roughness to the GISAXS intensity maps. A typical GISAXS map measured on the surface of a (Ge+SiO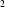)/SiO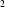 multilayer.

The third effect which should be considered is the roughness of the surface and interfaces in the modelled system. However, the reciprocal-space distribution scattered from the surface and/or interface roughness is usually concentrated in a relative stripe parallel to the 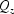 axis. This is demonstrated in Fig. 19 ▶, which shows the GISAXS map measured on a rough surface of a multilayer without quantum dots. The width of the intensity stripe along 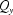 is approximately 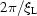, where 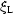 is the lateral correlation length of the interface roughness (see Pietsch *et al.*, 2004 ▶). Therefore, if this correlation length is larger than the mean separation of the dots, the contribution of the roughness can easily be distinguished. If 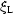 is comparable to the dot separation, the problem is more complicated and a detailed comparison of the experimental GISAXS data with simulations (including the roughness effect) must be performed.

The surface and interface effets are well known, so we will not consider them here (see Pietsch *et al.*, 2004 ▶).

## 5. Conclusion

We have developed theoretical models for the description of GISAXS intensity distributions from various types of three-dimensional QD lattices. The lattice types differ in the type of QD ordering and in the degree of disorder. The models are supported with experimental examples showing applications of the models to real systems. The structural parameters obtained from the GISAXS analysis using the developed models are in excellent agreement with the parameters obtained by microscopic measurement. The developed models can be applied to a wide variety of QD systems and they enable precise determination of QD lattice type, its parameters, disorder type and degree of disorder, as well as QD size and size distribution parameters.

## Figures and Tables

**Table 1:** Sets of parameters (P1–P12) used for the simulations of the GISAXS intensity maps The QD lattice is assumed to be rhombohedral with the basis vectors 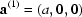, 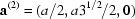, 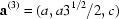, the dot radius is 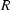 = 2.0 nm, with the r.m.s. deviation 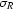 = 0.45 nm (Gamma distribution was used), the number of layers is 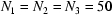. All values are in nm.

Parameters	*a*	*c*	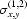	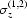	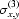	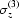
P1	10.0	10.0	1.5	1.0	1.0	1.0
P2	10.0	10.0	2.0	1.0	1.0	1.0
P3	10.0	10.0	2.5	1.0	1.0	1.0
P4	10.0	10.0	1.0	1.5	1.0	1.0
P5	10.0	10.0	1.0	2.0	1.0	1.0
P6	10.0	10.0	1.0	2.5	1.0	1.0
P7	10.0	10.0	2.5	1.0	1.5	1.0
P8	10.0	10.0	2.5	1.0	2.0	1.0
P9	10.0	10.0	2.5	1.0	2.5	1.0
P10	10.0	10.0	2.5	1.0	1.0	1.2
P11	10.0	10.0	2.5	1.0	1.0	1.4
P12	10.0	10.0	2.5	1.0	1.0	1.6

**Table 2:** Sets of parameters obtained by fitting the experimentally measured GISAXS intensity maps and determined from STEM cross sections for examples 1–3 The basis vectors are given by 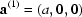, 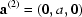, 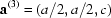 for example 1, and 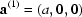, 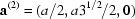, 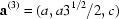 for examples 2 and 3. All values are given in nm except 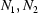 and 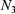, which show the number of QDs along basis vectors 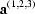, respectively. The number in brackets indicates the statistical error of the parameter.

	Example 1		Example 2		Example 3	
Parameter	GISAXS	STEM	GISAXS	STEM	GISAXS	STEM
*a*	8.6 (0.5)	9 (2)	21.4 (0.5)	21.0 (4)	12.3 (0.5)	12 (3)
*c*	6.9 (0.3)	7 (1)	14.0 (0.3)	14 (1)	12.8 (0.3)	12 (1)
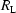	1.6 (0.1)	1.6 (0.5)	4.1 (0.1)	4.1 (0.5)	2.9 (0.1)	2.9 (0.5)
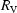	3.6 (0.3)	3.6 (0.5)	4.1 (0.1)	4.1 (0.5)	2.9 (0.1)	2.9 (0.5)
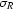	0.4 (0.1)		1.3 (0.3)		0.4 (0.1)	0.4
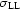	2.3 (0.2)		8.6 (0.4)		3.8 (0.2)	
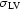	2.0 (0.2)		1.1 (0.2)		0.2 (0.1)	
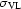	0.9 (0.1)		8.0 (0.2)		3.3 (0.2)	
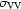	1.5 (0.3)		2.6 (0.2)		0.8 (0.1)	
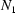	60 (20)		60 (20)		60 (20)	
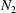	60 (20)		60		60 (20)	
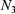	20 (0)	20 (0)	20 (0)	20 (0)	20 (0)	20 (0)
